# Factors associated with low birth weight: a case- control study

**DOI:** 10.15649/cuidarte.5511

**Published:** 2026-05-04

**Authors:** Daniela Alejandra Getial Mora, Leidys Paola Aguirre Contreras, Maritza Afanador Cataño, Juan Camilo Tocora Rodríguez

**Affiliations:** 1 Fundación Universitaria del Área Andina, School of Health and Sports Sciences. Master's Program in Epidemiology, Bogotá, Colombia. E-mail: dgetial2@estudiantes.areandina.edu.co Fundación Universitaria del Área Andina Bogotá Colombia dgetial2@estudiantes.areandina.edu.co; 2 Fundación Universitaria del Área Andina, School of Health and Sports Sciences. Master's Program in Epidemiology, Bogotá, Colombia. E-mail: laguirre71@estudiantes.areandina.edu.co Fundación Universitaria del Área Andina Bogotá Colombia laguirre71@estudiantes.areandina.edu.co; 3 Fundación Universitaria del Área Andina, School of Health and Sports Sciences. Master's Program in Epidemiology, Bogotá, Colombia. E-mail: mafanador4@estudiantes.areandina.edu.co Fundación Universitaria del Área Andina Bogotá Colombia mafanador4@estudiantes.areandina.edu.co; 4 Fundación Universitaria del Área Andina. School of Health and Sports Sciences. Master's Program in Epidemiology, Bogotá, Colombia. E-mail: jtocora2@areandina.edu.co Fundación Universitaria del Área Andina Bogotá Colombia jtocora2@areandina.edu.co

**Keywords:** Infant, Low Birth Weight, Term Birth, Risk Factors, Maternal Health, Recién Nacido de Bajo Peso, Nacimiento a Término, Factores de Riesgo, Salud Materna, Recém-Nascido de Baixo Peso, Nascimento a Termo, Fatores de Risco, Saúde Materna

## Abstract

**Introduction:**

Term low birth weight is a multifactorial event influenced by maternal, fetal, and socio-environmental conditions. Understanding it enables the design of targeted preventive interventions.

**Objective:**

To analyze the factors associated with low birth weight in term newborns attended at a tertiary-level clinic in Córdoba between 2020 and 2023.

**Materials and Methods:**

A retrospective case–control study. The sample size was calculated with 80% power and a 95% confidence level. A total of 84 cases and 169 controls were included through stratified random sampling. Maternal clinical and sociodemographic variables were analyzed using multivariate logistic regression.

**Results:**

Low birth weight was associated with hospitalization during pregnancy (aOR = 6.05; 95% CI: 2.94–12.47), rural residence (aOR = 2.65; 95% CI: 1.21–5.81), and female sex of the newborn (aOR = 1.97; 95% CI: 1.07–3.64). Maternal reproductive tract surgery acted as a protective factor (aOR = 0.37; 95% CI: 0.20–0.71).

**Discussion:**

The findings highlight the combined influence of clinical and social factors, particularly gestational hospitalization and rural residence, conditions that may limit timely prenatal care and increase perinatal risk.

**Conclusion:**

Preventing low birth weight requires strengthening the quality of prenatal care, prioritizing follow-up for pregnant women with prior hospitalization, and establishing differentiated care pathways for rural women to improve access and ensure timely maternal–fetal care.

## Resumen

**Introducción:**

El bajo peso al nacer a término es un evento multifactorial influido por condiciones maternas, fetales y socioambientales. Su comprensión permite orientar intervenciones preventivas.

**Objetivo:**

Analizar los factores asociados al bajo peso al nacer en recién nacidos a término atendidos en una clínica de tercer nivel en Córdoba entre 2020 y 2023.

**Materiales y métodos:**

Estudio de casos y controles retrospectivo. El tamaño muestral se calculó con potencia del 80% y un nivel de confianza del 95%. Se incluyeron 84 casos y 169 controles mediante muestreo aleatorio estratificado. Se analizaron variables maternas clínicas y sociodemográficas con regresión logística multivariada.

**Resultados:**

El bajo peso al nacer se asoció con hospitalización durante la gestación (ORa = 6,05; IC 95%: 2,94–12,47), residencia rural (ORa = 2,65; IC 95%: 1,21–5,81) y sexo femenino del recién nacido (ORa = 1,97; IC 95%: 1,07–3,64). La cirugía del aparato reproductor materno actuó como factor protector (ORa = 0,37; IC 95%: 0,20–0,71).

**Discusión:**

Los hallazgos evidencian la influencia combinada de factores clínicos y sociales, especialmente la hospitalización gestacional y la residencia rural, condiciones que pueden limitar la oportunidad del control prenatal y aumentar el riesgo perinatal.

**Conclusiones:**

La prevención del bajo peso al nacer exige fortalecer la calidad del control prenatal, priorizar el seguimiento de gestantes con antecedentes de hospitalización y establecer rutas de atención diferenciadas para mujeres rurales, con el fin de mejorar el acceso y garantizar un cuidado materno-fetal oportuno.

## Resumo

**Introdução:**

O baixo peso ao nascer a termo é um evento multifatorial influenciado por condições maternas, fetais e socioambientais. A compreensão desses fatores permite o desenvolvimento de intervenções preventivas.

**Objetivo:**

Analisar os fatores associados ao baixo peso ao nascer em recém- nascidos atermo atendidos em uma clínica terciária em Córdoba entre 2020 e 2023.

**Materiais e métodos:**

Estudo caso-controle retrospectivo. O tamanho da amostra foi calculado com poder de 80% e nível de confiança de 95%. Oitenta e quatro casos e 169 controles foram incluídos por meio de amostragem aleatória estratificada. As variáveis clínicas e sociodemográficas maternas foram analisadas por meio de regressão logística multivariada.

**Resultados:**

O baixo peso ao nascer foi associado à hospitalização durante a gravidez (OR ajustada = 6,05; IC 95%: 2,94–12,47), residência em área rural (OR ajustada = 2,65; IC 95%: 1,21–5,81) e sexo feminino do recém-nascido (OR ajustada = 1,97; IC 95%: 1,07–3,64). A cirurgia do sistema reprodutivo materno atuou como fator de proteção (OR ajustada = 0,37; IC 95%: 0,20–0,71). IC 95%: 0,20–0,71).

**Discussão:**

Esses achados demonstram a influência combinada de fatores clínicos e sociais, especialmente a hospitalização gestacional e a residência em área rural, condições que podem limitar o acesso ao pré-natal e aumentar o risco perinatal.

**Conclusões:**

A prevenção do baixo peso ao nascer requer o fortalecimento da qualidade do pré-natal, a priorização do acompanhamento de gestantes com histórico de hospitalização e o estabelecimento de fluxos de atendimento diferenciados para mulheres em área rural, a fim de melhorar o acesso e garantir o cuidado materno-fetal oportuno.

## Introduction

Low birth weight at term (LBW) refers to newborns who, despite completing between 37 and 41 weeks of gestation, are born weighing less than 2,500 grams^1^. This condition is a significant indicator of perinatal health risk, as it is closely associated with neonatal morbidity and mortality, as well as long-term consequences such as growth retardation, impaired cognitive development, and a higher likelihood of chronic diseases in adulthood^2^-^4^. According to the World Health Organization (WHO), newborns with LBW have up to 14 times the risk of neonatal death. Although a 30% reduction in its prevalence was proposed for 2025, global progress has been insufficient, and the figure remains close to 15%, so the target has been extended to 2030^1^,^2^,^5^.

Globally, it is estimated that between 15% and 20% of births are LBW, concentrated in low- and middle-income countries^6^. Colombia is no exception to this problem: the national proportion of LBW reached a record high of 11.3% in 2024^7^, with higher prevalence in urban areas and a clear inverse relationship with the multidimensional poverty index^1^,^8^. Despite advances in maternal and child health, significant gaps persist between regions, and it has been documented that departments with higher poverty levels have a greater burden of LBW^8^. According to recent studies in the Colombian population, the national prevalence ranges between 8% and 11%, confirming the role of clinical and social determinants in this outcome^9^.

These inequalities are also evident in other Latin American countries with similar socioeconomic characteristics, where recent research has shown that low maternal education, inadequate prenatal care, primiparity, and the presence of comorbidities such as anemia or hypertension increase the risk of LBW, even in term births^10^-^12^ . However, most of these studies come from large urban centers or regions with better living conditions, which limits the understanding of how clinical and social determinants interact in territories with high structural poverty.

In the department of Córdoba, available records reflect a worrying situation. The prevalence of LBW reached 9.4% in 2022, with 646 reported cases, positioning the department as one of the most affected in the Caribbean region^13^. This condition is exacerbated by the marked territorial differences in access to health services, the predominance of unfavorable socioeconomic conditions, and the structural vulnerability of the population using regional referral institutions^14^,^15^,^16^. In particular, the high-complexity clinic where this study is being conducted serves a diverse population from across the department and surrounding areas, making it a strategic point for understanding the determinants of LBW in contexts of high inequality.

In summary, although LBW has been extensively studied, a gap persists in recent and specific evidence in Córdoba that integrates clinical and sociodemographic factors within a context of high health inequity. Generating this information is essential for designing context-specific interventions, improving the early identification of at- risk pregnant women, strengthening perinatal care, and guiding effective public policies in maternal and child health.

In this context, the following research question arises: what are the sociodemographic and clinical factors associated with low birth weight in full-term newborns, treated between 2020 and 2023 in a third-level institution in the department of Córdoba?

## Materials and Methods

**Design and population**

This was an observational, analytical, retrospective case-control study. It was conducted at a tertiary care clinic in Córdoba, Colombia, which serves the population of the entire Colombian Caribbean region. The source population consisted of births registered between 2020 and 2023 in the National Registry of Affiliates- Births and Deaths (RUAF-ND).

**Eligibility criteria**

Term newborns (≥37 weeks) with complete medical records were included. Cases were neonates weighing ≤2,500 g and controls weighing between 2,501 and 4,000 g. Congenital malformations, multiple gestations, and maternal weight <40 kg were excluded due to the risk of confounding associated with severe malnutrition.

**Sample size and sampling**

**Figure 1:**
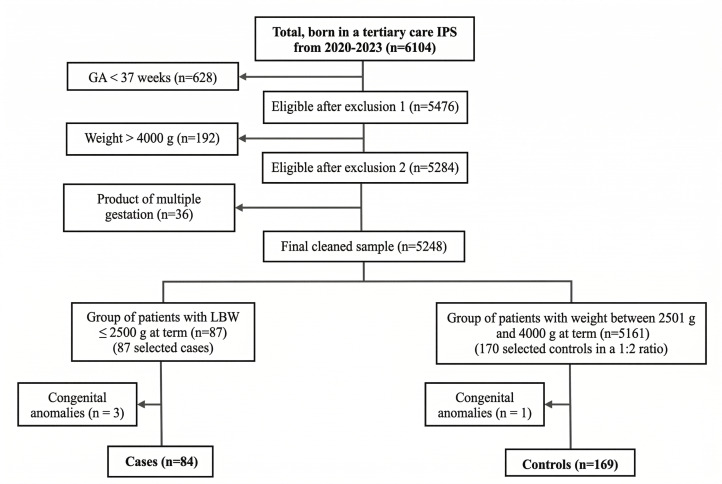
Flowchart of the sample selection process

*Note: IPS: Healthcare Provider Institution (IPS, by its Spanish acronym); GA: gestational age; LBW: low birth weight.*

Sample size was calculated using Epidat 4.2 (95% CI, 80% power), based on a reference exposure to a history of threatened preterm labor of 8.6% in cases and 1.1% in controls. An estimated 85 cases and 170 controls were needed^17^. After applying selection criteria, the final sample consisted of 84 cases and 169 controls. Controls were selected using stratified random sampling by gestational age (37, 38, 39, and ≥40 weeks). Within each stratum, controls were randomly selected using the random selection module of IBM SPSS Statistics version 29 (institutionally licensed), ensuring impartial and reproducible allocation. The process is summarized in Figure 1.

**Variables**

The dependent variable was LBW (≤2,500g). Independent variables included sociodemographic factors (maternal age, education level, marital status, affiliation with the Colombian General System of Social Security in Health, area of residence) and clinical factors (parity, number of prenatal visits, gestational hypertension, gestational diabetes, maternal anemia, urinary tract infection, type of delivery). These were operationalized according to institutional clinical definitions and national guidelines, as categorical or continuous variables depending on their nature. Data were obtained from the RUAF-ND (National Registry of Affiliates - Births and Deaths) and electronic health records.

Variables associated with both exposure and outcome were considered as possible confounding factors, including maternal age, educational level, health system affiliation regime, number of prenatal check-ups, and the presence of maternal pathologies (anemia, hypertension, or gestational diabetes).

**Bias control**

Strict data selection and verification criteria were applied. The database was reviewed for consistency and missing values. In cases with inconsistent or incomplete information, a direct comparison was made with the electronic health record before final inclusion in the database. Confounding bias was controlled using multivariate logistic regression. Variables with p < 0.25 or clinical relevance were included in the model; those without significant contribution were excluded for parsimony.

**Statistical analysis**

Frequencies, means, or medians were used depending on the distribution (Kolmogorov-Smirnov). For bivariate analysis, Chi-square or Fisher's exact tests and Student's t-test or Spearman's rank correlation coefficient were applied. Crude odds ratios with 95% confidence intervals were calculated. The multivariate model was fitted using binary logistic regression, evaluating collinearity (VIF>5) and Hosmer-Lemeshow^18^ goodness of fit and discriminative capacity using the area under the ROC curve (AUC) (considering ≥0.70 adequate). The data were stored in Mendeley Data^19^.

**Ethical aspects**

This research was classified as low risk according to Resolution 8430 of 1993 of the Colombian Ministry of Health^20^. Approval was obtained from the Ethics Committee of Clínica Zayma SAS and Universidad Área Andina. Confidentiality and anonymity were guaranteed through the removal of personal data and restricted access to the database.

## Results

A total of 253 pregnant women with full-term newborns who attended a tertiary care clinic in the department of Córdoba between 2020 and 2023 were analyzed. Of these, 84 (33.20%) comprised the case group LBW and 169 (66.79%) the control group (newborns with appropriate birth weight). The median maternal age was 24 years (IQR: 20–30) in the case group and 26 years (IQR: 21–30) in the control group. Most pregnant women resided in urban areas (72.62% in cases; 86.39% in controls), while a higher proportion of cases (27.38%) resided in rural areas compared to controls (13.60%). In both groups, most pregnant women were enrolled in the contributory health insurance system (67.86% in cases and 74.56% in controls). Differences were observed in variables such as areas of residence, education level, and marital status. Sociodemographic characteristics are detailed in Table 1. 

**Table 1:** Sociodemographic characteristics by study group (2020–2023)

Sociodemographic variables	Cases (n=84) % (n)	Controls (n=169) % (n)	p-value
Area of residence			0.007
Rural	27.38 (23)	13.61 (23)	
Urban	72.62 (61)	86.39 (146)	
Marital status			0.985
Single woman	8.33 (7)	8.28 (14)	
Common-law union	65.48 (55)	64.50 (109)	
Married	26.19 (22)	27.2 2 (46)	
Membership scheme			0.354
Subsidized	30.95 (26)	25.44 (43)	
Contributory	69.05 (58)	74.56 (126)	
Occupation			0.706
Unemployed	5.95 (5)	9.47 (16)	
Housewife	36.90 (31)	35.50 (60)	
Student	1 1.90 (10)	8.88 (15)	
Employee	45.24 (38)	46.15 (78)	
Education			0.166 *
Incomplete primary education	4.76 (4)	13.02 (22)	
Completed primary education	13.10 (11)	11.83 (20)	
Baccalaureate	33.33 (28)	32.54 (55)	
Technical	17.86 (15)	8.88 ( 15)	
University	27.38 (23)	29.59 (50)	
Postgraduate	3.57 ( 3)	4.14 (7)	

* Fisher's exact test, when expected values were <5.

Regarding clinical characteristics, significant differences were observed between the groups. Pregnant women in the case groups presented a higher frequency of hypertensive disorders of pregnancy (40.47% vs. 17.75% p <0.001), history of gestational anemia (17.86% vs. 4.14%, p <0.001), urinary tract infections (20.24% vs. 7.10%, p = 0.002), risk of miscarriage (10.71% vs. 3.55%, p = 0.026), amniotic fluid abnormalities (21.43% vs. 5.92%, p <0.001), hospitalization during pregnancy (41.67% vs. 10.06%, p <0.001), and history of reproductive system surgery (27.38% vs. 49.70%, p = 0.001). Furthermore, a higher proportion of female newborns was identified in the case group (67.86% vs. 49.70%, p = 0.006). The other clinical variables did not show statistically significant differences between the groups. Clinical characteristics are detailed in Table 2.

Quantitative variables were compared between the case and control groups. Maternal age, which was normally distributed, was reported as mean ± standard deviation and showed no statistically significant differences between the groups (28.11 ± 7.13 vs. 28.81 ± 6.07 years; p = 0.439). In contrast, the other variables were not normally distributed and were therefore reported as median and interquartile range. Birth weight was considerably lower in the case group, with a median of 2,325 g compared to 3,090 g in the controls (p = 0.001), as expected. Furthermore, pregnant women in the case group had a lower number of live births (median 1 vs. 2; p = 0.005) and a significant difference in the number of pregnancies (p = 0.016). The other variables did not show statistically relevant differences. The results are summarized in Table 3. 

**Table 2:** Clinical characteristics by study group (2020–2023)

Clinical variables	Cases (n=84) % (n)	Controls (n=169) % (n)	p-value
Type of delivery			0.985
Caesarean section	84.52 (71)	84.62 (143)	
Vaginal delivery	15.48 (13)	15.38 (26)	
Sex of the newborn			0.006
Female	67.86 (57)	49.70 (84)	
Male	32.14 (27)	50.30 (85)	
History of chronic illnesses			0.055 *
High blood pressure	3.57 (3)	1.18 (2)	
Obesity	0.00 (0)	2.96 (5)	
Autoimmune disease	0.00 (0)	1.18 (2)	
Chronic respiratory disease	2.38 (2)	1.18 (2)	
Mental illness	3.57 (3)	0.59 (1)	
Cardiovascular disease	1.19 (1)	0.59 (1)	
Thyroid disease	3.57 (3)	1.78 (3)	
Obesity with bariatric surgery	2.38 (2)	0.00 (0)	
Other	2.38 (2)	5.92 (10)	
None	80.95 (68)	84.62 (143)	
Hypertensive disorders of pregnancy			0.001 *
Gestational hypertension	26.19 (22)	14.79 (25)	
Preeclampsia	9.52 (8)	2.96 (5)	
Severe preeclampsia	2.38 (2)	0.00 (0)	
HELLP syndrome	1.19 (1)	0.00 (0)	
Chronic hypertension with preeclampsia Over-added	1.19 (1)	0.00 (0)	
None	59.52 (50)	82.25 (139)	
Risk of miscarriage			0.026
Yes	10.71 (9)	3.55 (6)	
No	89.29 (75)	96.45 (163)	
Urinary tract infections			0.002
Yes	20.24 (17)	7.10 (12)	
No	79.76 (67)	92.90 (157)	
Gestational diabetes			1.001*
Yes	1.19 (1)	1.78 (3)	
No	98.81 (83)	98.22 (166)	
Gestational anemia			0.001
Yes	17.86 (15)	4.14 (7)	
No	82.14 (69)	95.86 (162)	
Amniotic fluid abnormalities			0.001 *
Oligohydramnios	21.43 (18)	4.73 (8)	
Polyhydramnios	0.00 (0)	1.18 (2)	
None	78.57 (66)	94.08 (159)	
Reproductive system surgery			0.001
Yes	27.38 (23)	49.70 (84)	
No	72.62 (61)	50.30 (85)	
Hospitalization during pregnancy			0.001
Yes	41.67 (35)	10.06 (17)	
No	58.33 (49)	89.94 (152)	
Toxoplasmosis			0.336 *
Yes	3.57 (3)	1.18 (2)	
No	96.43 (81)	98.82 (167)	
Obstetric risk classification			0.095
High	89.29 (75)	81.07 (137)	
Low	10.71 (9)	18.93 (32)	
Gynecological pathologies			0.107 *
Uterine alteration	9.52 (8)	10.06 (17)	
Vaginal infection	7.14 (6)	3.55 (6)	
Vaginal canal trauma	3.57 (3)	0.00 (0)	
Other	2.38 (2)	2.96 (5)	
None	77.38 (65)	83.43 (141)	
Interpregnancy period			1.000*
Short	1.19 (1)	2.37 (4)	
Intermediate	19.05 (16)	24.26 (41)	
Long	26.19 (22)	32.54 (55)	
Not applicable	48.81 (41)	26.63 (45)	
No data	4.76 (4)	14.20 (24)	

* Fisher's exact test, when expected values were <5.

**Table 3:** Quantitative characteristics by study group (2020–2023)

Variable	Cases (n=84) Med [IQR]	Controls (n=169) Med [IQR]	p-value
Maternal age (years) M±SD	28.11 ± 7.13	28.81 ± 6.07	0.439
Number of pregnancies	2 [1;2]	2 [1;3]	0.016
Number of pregnancy losses	0 [0;0]	0 [0;1]	0.737
Number of live births	1 [1;2]	2 [1;3]	0.005
Number of prenatal checkups	7.5 [6;8]	7 [6.5;8]	0.714
Gestational age (weeks)	37 [37;38]	37 [37;38]	0.808

M: mean; SD: standard deviation; Student's t-test with Welch's correction (Levene's test: p = 0.042). Med: median; IQR: interquartile range; Mann-Whitney U test.

In the multiple logistic regression, the variables that remained in the final model were rural residence (OR: 2.649; 95% CI: 1.209–5.806), female sex of the newborn (OR: 1.970; 95% CI: 1.065–3.642), hospitalization during pregnancy (OR: 6.052; 95% CI: 2.937–12.473), and reproductive system surgery (OR: 0.374; 95% CI: 0.196–0.712), the latter as a protective factor. Maternal age remained as a continuous variable, although it did not reach statistical significance (OR: 0.960; 95% CI: 0.912–1.010). In contrast, some clinical factors that showed a significant association in the bivariate analysis were not included due to insufficient frequencies, which prevented meeting the assumptions of logistic regression, or because they were collinear with variables of greater clinical relevance, especially hospitalization during pregnancy and hypertensive disorders of pregnancy. Furthermore, the parsimony criterion was applied, retaining only those variables with an independent and stable contribution to predicting the outcome. The complete results of the model are presented in Table 4.

**Table 4:** Factors associated with low birth weight (crude and adjusted ORs)

Variable	OR crude (95% CI)	p-value	OR adjusted (95% CI)	p-value
Area of residence		0.007		0.015
Urban	1		1	
Rural	2.39 (1.24 – 4.58)		2.64 (1.20 – 5.80)	
Sex of the newborn		0.006		0.031
Male	1		1	
Female	2.13 (1.23 – 3.69)		1.97 (1.06 – 3.64)	
Urinary tract infections		0.002		0.099
No	1		1	
Yes	3.32 (1.50 – 7.33)		2.27 (0.85 – 6.06)	
Hospitalization during pregnancy		0.001		0.001
No	1		1	
Yes	6.38 (3.29 – 12.39)		6.05 (2.93 – 12.47)	
Reproductive system surgery		0.001		0.003
No	1		1	
Yes	0.38 (0.21 – 0.67)		0.37 (0.19 – 0.71)	
Gestational anemia		0.001		
No	1		—	—
Yes	5.54 (2.14 – 13.83)			
Risk of miscarriage		0.026		
No	1		—	—
Yes	2.65 (1.09 – 6.41)			
Maternal age (continued)	—	—	0.96 (0.91 – 1.01)	0.409

OR: odds ratio; 95% CI: 95% confidence interval. —: Not applicable or variable not included in the fitted model.

The final model showed adequate overall fit, according to the Hosmer- Lemeshow test (p > 0.05), and presented acceptable discriminative capacity, with an AUC of 0.78, (SE: 0.030; 95% CI: 0.729–0.847; p < 0.001), which indicates adequate performance in identifying pregnant women with a higher probability of having newborns with LBW. 

## Discussion

The results of the binary logistic regression model identified independent factors associated with LBW. Pregnant women residing in rural areas were more likely to have a newborn with LBW, reinforcing evidence of territorial inequalities in access to and quality of prenatal care. The female sex of the newborn was also found to be associated with a higher risk of LBW, suggesting that even non-modifiable biological factors can influence perinatal outcomes. Hospitalization during pregnancy was the strongest association, indicating that severe clinical complications during pregnancy pose a significant risk to fetal development, possibly through mechanisms such as hypoxia, systemic inflammation, or placental restriction. In contrast, a history of reproductive tract surgery showed an inverse association with LBW, possibly related to greater adherence to prenatal care in these patients.

However, it would be advisable to analyze in greater detail the variables that lost significance in the adjusted model, such as anemia and urinary tract infections, since, despite not showing an independent statistical association, they may represent clinically relevant factors whose effect could be mediated by other obstetric conditions or limited by the frequency of events. These types of variables remain epidemiologically important and should be considered in perinatal risk surveillance.

The finding related to rural residence coincides with studies conducted in Ethiopia, where it was observed that women living in rural areas were approximately three times more likely to have LBW infants compared to urban women^21^. In these contexts, pregnant women face geographic and economic barriers to accessing health services, including early initiation of prenatal care, nutritional monitoring, and timely detection of warning signs. In the Colombian context, these gaps may be exacerbated by the unequal distribution of high-complexity services and by limitations in the timeliness of medical transport, which could partially explain the persistence of adverse perinatal outcomes in rural areas even when acceptable insurance coverage exists.

Regarding the sex of the newborn, the results showed a higher probability of LBW in girls than in boys. This pattern has been observed in previous studies, such as research conducted in Brazil, which reported a higher proportion of LBW in female newborns, with a magnitude of association like that found in this study^22^. It has been proposed that this difference may be related to a slower rate of fetal growth in girls, influenced by hormonal, genetic, and possibly epigenetic factors^23^. Furthermore, reviews conducted in South America have documented a higher rate of intrauterine growth restriction in newborns, even in the absence of maternal comorbidities^24^. However, given that this is a non-modifiable biological characteristic, these findings should be interpreted with caution and, rather than a direct target for intervention, serve as a tool for risk stratification and prioritizing fetal growth monitoring.

Hospitalization during pregnancy was the factor most strongly associated with LBW, suggesting that it may act as a marker of high-risk obstetric conditions rather than a direct causal factor^25^. This finding is consistent with previous research that has shown a higher risk of LBW in pregnant women hospitalized for complications such as gestational hypertension, preeclampsia, or severe infections^25^. In this context, hospitalization should not be considered a causal factor, but rather an indicator of clinical severity, highlighting the importance of specialized monitoring protocols for pregnant women requiring inpatient care.

A particularly interesting finding was the inverse association between a history of reproductive system surgery and the occurrence of LBW. Although this result is poorly documented in the regional literature, it could be related to a higher frequency of specialized checkups in these patients. It is possible that women with a history of gynecological surgery maintain a closer connection with the healthcare system, which would facilitate access to more rigorous and timely prenatal care. Recent research has suggested that this history may promote greater adherence to medical care during pregnancy^26^, although further studies are needed to confirm this hypothesis and explore its applicability in other contexts.

Regarding urinary tract infections (UTIs) during pregnancy, a trend toward statistical significance was observed, although it was not reached in the adjusted model. Even so, the direction of the effect suggests a possible clinically relevant relationship. The literature has extensively documented that UTIs can increase the risk of preterm birth and LBW by inducing systemic inflammatory responses and altering placental function^27^. A systematic review published in 2023 reported a prevalence of 7.5% for symptomatic UTIs in pregnant Latin American women and their association with adverse outcomes^27^. Although the findings of the present study do not allow for establishing an independent association, the observed trend justifies its follow-up in future research, especially in populations with limited access to health services and in designs that allow for better measurement of the infectious burden and timely treatment.

In the bivariate analysis, motherhood in women under 18 years of age was associated with a higher risk of LBW; however, this variable was not included in the adjusted model due to the low frequency of cases.

Studies conducted in other regions have found a higher probability of LBW in adolescents, associated with conditions such as biological immaturity, malnutrition, lower adherence to prenatal care, and a context of social vulnerability^28^,^29^. While this result should be interpreted with caution, it highlights the need to continue investigating the impact of young mothers on perinatal outcomes in vulnerable populations and to design specific sexual and reproductive health interventions aimed at adolescents, which could have an indirect effect on reducing LBW.

The findings of this study provide local evidence on factors associated with LBW, integrating clinical, biological, and social elements. The inclusion of non-modifiable variables such as fetal sex, along with clinical factors such as gestational hospitalization and social determinants such as rural residence, allows for a broader understanding of the phenomenon and offers useful elements for planning targeted interventions. In particular, the results suggest the need to strengthen the early identification and intensive follow-up of hospitalized pregnant women and women residing in rural areas, as well as to optimize referral and counter-referral pathways between levels of care.

**Strengths and limitations**

This study has several strengths. The 1:2 case-control design increased statistical power and allowed for the evaluation of multiple factors associated with LBW. The use of multivariable models improved internal validity by adjusting for relevant confounders, and the cleaning of clinical records strengthened the quality of the information.

Among the limitations, the retrospective nature of the study may introduce information bias, as it relies on clinical records with potential omissions or variability in measurement. Selection bias is also present, since the study was conducted at a single tertiary care center that primarily serves high-risk pregnancies, limiting its generalizability to rural or primary care populations. Furthermore, the absence of key social variables may leave residual confounding. As with all case-control studies, the findings describe associations and do not allow for inferences of causality.

Even so, the results provide useful evidence for practice and the organization of services. They support the need to strengthen the quality of prenatal care, prioritize pregnant women with clinical and social vulnerabilities, and establish differentiated care pathways, especially for rural women, as part of maternal and perinatal health policies.

## Conclusions

The study confirmed that LBW remains a significant problem influenced by clinical and social determinants such as rural residence, female sex, gestational hospitalization, and a history of reproductive system surgery. These findings support the need to strengthen risk screening upon entry into prenatal care, implement differentiated pathways for pregnant women in rural areas, and ensure fetal growth monitoring in accordance with the WHO's package of prenatal care. At the tertiary level, it is recommended to optimize referral and counter-referral criteria, improve coordination for medical transport, and expand the use of telemedicine for follow-up. Future research should evaluate interventions adapted to rural areas and explore factors not included in the study, such as maternal nutrition or socioeconomic conditions, to broaden our understanding of the phenomenon.
